# The effect of common medications on the efficacy of immune checkpoint inhibitors

**DOI:** 10.1002/cncr.70222

**Published:** 2025-12-14

**Authors:** Daria Brinzevich, Virginia Falvello, Sadiq S. Rehmani, Garth W. Strohbehn, Nithya Ramnath, Michael P. Dykstra, Luke M. Higgins, David Elliott, Matthew J. Schipper, Michael D. Green, Alex K. Bryant

**Affiliations:** ^1^ Division of Hematology/Oncology Department of Medicine University of Michigan Ann Arbor Michigan USA; ^2^ Veterans Affairs Center for Clinical Management Research Ann Arbor Michigan USA; ^3^ Division of Oncology LTC Charles S. Kettles VA Medical Center Ann Arbor Michigan USA; ^4^ Institute for Health Policy and Innovation University of Michigan Ann Arbor Michigan USA; ^5^ Lung Precision Oncology Program Charles S. Kettles VA Medical Center Ann Arbor Michigan USA; ^6^ Rogel Cancer Center Michigan Medicine Ann Arbor Michigan USA; ^7^ Department of Radiation Oncology Veterans Affairs Ann Arbor Healthcare System Ann Arbor Michigan USA; ^8^ Department of Radiation Oncology University of Michigan Ann Arbor Michigan USA; ^9^ Department of Biostatistics University of Michigan Ann Arbor Michigan USA; ^10^ Anchorage Radiation Oncology Center Anchorage Alaska USA

**Keywords:** carcinoma, immune checkpoint inhibitors, immunotherapy, lung neoplasms, non‐small‐cell lung, Veterans Health

## Abstract

**Background:**

Medications such as proton pump inhibitors (PPIs), antihistamines, and nonsteroidal anti‐inflammatory drugs have been linked to immune checkpoint inhibitor (ICI) efficacy in patients with non–small cell lung cancer (NSCLC), but these associations may reflect unmeasured confounding rather than true pharmacologic effects. This study evaluated whether commonly prescribed medications influence ICI outcomes, using a national patient sample and a negative control cohort.

**Methods:**

The authors identified Veterans Health Administration (VHA) patients with stage IV NSCLC treated with first‐ or second‐line ICI therapy (*n* = 3739) or chemotherapy (*n* = 6585) from 2005 to 2023. Baseline use of 20 common medication classes and an immunomodulatory drug score were assessed. Propensity‐weighted Cox regression evaluated associations between each medication class and overall survival (OS) or time‐to‐next treatment (TTNT) in the ICI group. For any medication with a nominally significant association (*p* < .05), the same analysis was repeated in the chemotherapy group to test for nonspecific effects.

**Results:**

After propensity weighting, 14 of 20 medication classes showed no association with OS or TTNT in the ICI cohort. Loop diuretics, anticoagulants, opioids, penicillin antibiotics, and fluoroquinolone antibiotics were associated with worse outcomes, but similar effects were seen in the chemotherapy group. A higher immunomodulatory drug score was also associated with inferior outcomes among ICI patients, but this association was likewise present in the chemotherapy cohort.

**Conclusion:**

In this study, commonly prescribed medications did not appear to alter ICI efficacy in stage IV NSCLC. Prior associations reported in the literature may be attributable to unmeasured confounding rather than true drug–immunotherapy interactions.

## Abstract 2

In a large national Veterans Health Administration cohort, the study evaluated whether 20 common medication classes and an “immunomodulatory drug score” influence outcomes among patients with stage IV non–small cell lung cancer treated with immune checkpoint inhibitors (ICIs), using a chemotherapy cohort as a negative control and propensity‐weighted Cox models for overall survival and time‐to‐next‐treatment. Fourteen of 20 classes showed no association with ICI outcomes; apparent detriments for loop diuretics, anticoagulants, opioids, antibiotics, and a higher drug score were similarly prognostic in the chemotherapy cohort, suggesting no ICI‐specific medication effect.

## INTRODUCTION

Immune checkpoint inhibitors (ICIs) are a cornerstone of cancer therapy, with Food and Drug Administration (FDA) approval across more than 20 malignancies. Patients receiving ICIs often take chronic medications to manage cardiovascular, gastrointestinal, psychiatric, and other comorbid conditions. Preclinical studies suggest that some of these medications may modulate ICI efficacy by influencing immune pathways. For example, opioids have been associated with reduced CD8+ T‐cell tumor infiltration,^1^ whereas proton pump inhibitors (PPIs) have been shown to alter the gut microbiome in ways that may impair ICI response.^2^, ^3^ Other drugs—including nonsteroidal anti‐inflammatories (NSAIDs),^4^ β‐blockers,^5^ statins,^6^ angiotensin‐converting enzyme inhibitor(s) and angiotensin receptor blocker(s) (ACE‐I/ARBs),^7^ and metformin,^8^ among others—have been proposed to enhance ICI efficacy via immune‐stimulating mechanisms.

Clinical evidence for the impact of these medications on ICI efficacy is mixed. Although some observational studies and meta‐analyses have linked statin use to improved survival^9^ and PPIs to worse outcomes,^10^, ^11^, ^12^ others have found no effect.^11^, ^13^, ^14^ Similarly, studies across various cancers have reported null associations for NSAIDs, metformin, antipsychotics, β‐blockers, and H_2_ antagonists.^10^, ^15^, ^16^ These inconsistencies may reflect structural limitations in the existing literature: most studies are small, single‐center cohorts with limited ability to control for confounding by indication or the healthy user effect.^17^ For example, opioid users may have worse overall survival (OS) than nonusers due to extensive, symptomatic, disseminated cancer that requires more intensive pain control relative to nonusers with lower‐volume disease, a fact that is usually not captured by structured medical record data. Similarly, statin users may be generally healthier than nonusers in unmeasured ways due to the healthy user bias,^17^ leading to the appearance of improved OS without a true effect on ICI efficacy.

One strategy to address these biases is the use of negative control groups—patients for whom no biologic drug–ICI interaction is expected. Chemotherapy‐treated patients serve as an ideal control: if a medication is associated with worse survival in both ICI and chemotherapy cohorts, this suggests the drug is a general prognostic marker rather than an ICI‐specific modifier. Conversely, a differential effect confined to the ICI cohort would support a true pharmacologic interaction with ICIs. This helps distinguish true drug–ICI interactions from spurious associations.

In this study, we sought to address the prior literature’s weaknesses of small sample sizes, inadequate control for confounding, and a lack of negative controls. We focused on a single, common malignancy (de novo stage IV non–small cell lung cancer [NSCLC]) and used the Veterans Health Administration (VHA) national database to build a large, homogeneous cohort of patients receiving first‐ or second‐line ICI therapy. After extensive confounding control, we analyze the associations between 16 common medication classes and ICI efficacy, sequentially testing any significant findings in a negative control group of stage IV NSCLC patients undergoing non‐ICI therapies to differentiate true drug–ICI interactions from spurious or confounded associations.

## MATERIALS AND METHODS

### Data source

This study used electronic medical record data from the VHA Corporate Data Warehouse (CDW), which includes data for all veterans receiving care through VHA facilities nationwide. This study was approved by the Veterans Affairs Ann Arbor institutional review board. Health Insurance Portability and Accountability Act authorization was waived because the study analyzed retrospective data and involved minimal risk to participants.

### Cohort definitions

We queried CDW for all patients with newly diagnosed (de novo) stage IV NSCLC undergoing first‐ or second‐line systemic therapy in the national VHA system from 2000 to 2024. Date at diagnosis, stage at diagnosis, and tumor histology were obtained through VA Cancer Registry System data. The ICI cohort included patients undergoing a limited set of ICI monotherapies (pembrolizumab or nivolumab) or combination chemoimmunotherapy (pembrolizumab with either carboplatin/pemetrexed or carboplatin/paclitaxel) from 2015 (when ICIs were first FDA‐approved in NSCLC) to 2024. The historical negative control cohort included patients undergoing platinum‐based combination chemotherapy regimens or a limited set of monotherapy regimens (pemetrexed, docetaxel, gemcitabine, paclitaxel, or vinorelbine; see Table S1 for full list of included regimens and their frequencies) from 2005 to 2015. We excluded patients who were ever exposed to ICIs.

### Medication exposures

We used outpatient VA pharmacy and inpatient medication administration records to collect prescriptions for each of 20 commonly used medication classes. These medication classes were chosen because they either had previously reported associations with ICI efficacy or toxicity (statins, proton pump inhibitors [PPIs], H_2_ antagonists [H2As], selective serotonin or serotonin‐norepinephrine reuptake inhibitors [SSRIs/SNRIs], nonsteroidal anti‐inflammatories [NSAIDs], β‐blockers, ACE‐I/ARBs, metformin, opioids, and antibiotics) or because of their widespread use in the veteran population (antiplatelets, calcium channel blockers, loop diuretics, antipsychotics, aspirin, and anticoagulants). The specific drugs comprising each medication class are presented in Table S2. Patients were classified as “users” or “nonusers” based on the presence of an outpatient or inpatient prescription for each drug within 3 months before therapy initiation. In sensitivity analyses, this definition was varied to within 12 months before therapy initiation.

### Outcome measures and covariates

Our primary outcomes included overall survival (OS) and time‐to‐next treatment (TTNT). TTNT is a surrogate for radiographic tumor progression^18^ and correlates with OS among ICI‐treated non–small cell lung cancer patients.^19^, ^20^ OS was defined as the time from the index date (treatment initiation) until death; patients were censored at their last VHA follow‐up. TTNT was defined as the time from the index date to death or initiation of a new systemic therapy, and patients were censored at the time of last follow‐up.

We collected additional variables to account for any baseline differences in medication users and nonusers. We captured additional demographic information from CDW including marital status, sex, and geographic region. Baseline comorbidities were assessed in the year before the index date. Tumor PD‐L1 expression was extracted from pathology and hematology/oncology notes using a previously validated natural language processing algorithm.^21^ Charlson Comorbidity Index (CCI) was calculated based on ICD‐9/10 codes^22^; codes for malignancies were excluded from the CCI calculation. Body mass index (BMI) was calculated using the closest height and weight to the index date. Baseline chronic kidney disease (CKD) was calculated using the CKD‐EPI 2021 formula.^23^ Baseline chronic obstructive pulmonary disease (COPD) severity was assessed using the number of COPD exacerbations in the prior year. COPD diagnoses were assessed through ICD‐9/10 codes; exacerbations were identified through short‐term (<14 days) steroid and/or antibiotic prescriptions coinciding with a general COPD diagnosis code, or the presence of an ICD‐9/10 diagnosis code specific for COPD exacerbation.^24^ Smoking status was obtained through health factors data.^25^, ^26^ Baseline hypothyroidism was defined as either a high thyroid stimulating hormone and low free T4 or a levothyroxine prescription. Baseline autoimmune disease was assessed using ICD‐9/10 codes for one of 25 autoimmune conditions, as previously described.^27^ To capture baseline health care utilization, we quantified emergency department, hematology/oncology, mental health, and social work encounters in the prior year. Prior radiation therapy was ascertained through CPT codes for radiotherapy delivery.

### Immunomodulatory drug score

Use of antibiotics and systemic steroids before treatment has been associated with inferior ICI tumor response and survival outcomes.^28^ To assess whether these medications affect ICI efficacy in our cohort, we applied an established three‐tier immunomodulatory drug score to our cohort. This score was suggested to be both prognostic and predictive of ICI benefit in NSCLC,^29^, ^30^, ^31^, ^32^ although has rarely been investigated in cohorts receiving therapies other than ICIs.^29^ The drug score assigns points for recent usage (within 1 month of ICI initiation) of antibiotics (1 point), PPIs (1 point), and corticosteroids (2 points). The continuous score is then categorized into tiers (Good: score 0; Intermediate: score 1–2; Poor: score 3–4). We calculated this score in both ICI and chemotherapy cohorts based on antibiotic, steroid, and PPI prescriptions before the index date.

### Statistical analysis

We took a sequential testing approach to assess the effect of each medication class on our efficacy outcomes. Limiting our analysis to the ICI cohort, we first performed propensity weighting with inverse probability of treatment weights^33^ to balance medication users and nonusers on all measured baseline covariates. The target estimand was the average treatment effect among the treated. For each outcome, we then performed weighted univariable Cox regression with robust standard errors to obtain adjusted hazard ratios (aHR) for each medication and outcome (OS and TTNT). This procedure resulted in a set of putative associations between medication use and ICI outcomes among ICI‐treated patients. To test the specificity of these associations to ICI treatment, for each medication with a nominally significant *p* value (<.05) in the ICI cohort, we performed the same analyses in the chemotherapy cohort. Similar aHRs in the ICI and chemotherapy cohorts would suggest that the medication is a general prognostic factor and is not specifically associated with ICI treatment outcomes.

Propensity weighting was performed separately for each medication class. Weighting performance was assessed by standardized differences. The propensity weight model included age, marital status, gender, BMI, tumor histology, prior radiation, number of prior lines of therapy (0 or 1), CCI, year of diagnosis, smoking status, time from original cancer diagnosis to index date, COPD exacerbations, CKD, number of oncology, mental health, and social work visits in the prior year, baseline autoimmune disease, and baseline hypothyroidism. The propensity model for the ICI group additionally included tumor PD‐L1 expression. For analyses of the immunomodulatory drug score on OS and TTNT, we used multivariable Cox regression adjusting for all baseline covariates. The proportional hazards assumption was checked by visual inspection of scaled Schoenfeld residuals. Unadjusted survival estimates were generated by the Kaplan–Meier method. Median follow‐up time was estimated with the reverse Kaplan–Meier method. Missing values in covariates were coded with a separate “unknown” level. All analyses were performed with R v4.3.1 (R Core Team, Vienna, Austria). Follow‐up for all analyses was current through July 1, 2024.

## RESULTS

### Cohort characteristics

We identified 3739 patients with de novo metastatic NSCLC treated with ICIs and 6585 patients treated with chemotherapy (control cohort) (Table 1). Among ICI patients, 97% were male, 70% were non‐Hispanic White, 81% were between 60 and 79 years old, 62% had adenocarcinoma tumor histology, 30% had tumor PD‐L1 expression 50%–100%, 45% received ICI in combination with chemotherapy, and 71% were treated in the first line. The control group was similar to the ICI group in most baseline covariates including demographics lines of prior therapy, and time from diagnosis to treatment (Table 1) though they tended to be younger (24% aged 59 or less vs. 9.4% in ICI group) and have fewer comorbidities (58% with CCI 0–1 vs. 49% in ICI group).

**TABLE 1:** Characteristics of the sample.

Characteristic	Immunotherapy, *N* = 3739^a^	Chemotherapy, *N* = 6585^a^
Age group (years), No. (%)		
59 or less	351 (9.4)	1550 (24)
60 to 69	1377 (37)	3136 (48)
70 to 79	1644 (44)	1476 (22)
80 or higher	367 (9.8)	423 (6.4)
Sex, No. (%)		
Female	118 (3.2)	185 (2.8)
Male	3621 (97)	6400 (97)
Self‐reported race, No. (%)		
Non‐Hispanic White	2615 (70)	4599 (70)
Non‐Hispanic Black	787 (21)	1199 (18)
Hispanic	116 (3.1)	139 (2.1)
Other	62 (1.7)	76 (1.2)
Unknown	159 (4.3)	572 (8.7)
Region, No. (%)		
Continental	532 (14)	1260 (19)
Midwest	1086 (29)	1694 (26)
North Atlantic	856 (23)	1613 (24)
Pacific	581 (16)	781 (12)
Southeast	684 (18)	1237 (19)
Year of diagnosis, No. (%)		
2001–2005	0 (0)	449 (6.8)
2006–2010	0 (0)	3128 (48)
2011–2015	2 (<0.1)	3008 (46)
2016–2020	2424 (65)	0 (0)
2021–2024	1313 (35)	0 (0)
Marital status, No. (%)		
Divorced	1129 (30)	2109 (32)
Married	1802 (48)	3039 (46)
Never married	394 (11)	557 (8.5)
Other	414 (11)	880 (13)
BMI group, No. (%)		
Healthy weight	1564 (42)	2977 (45)
Obese	711 (19)	1035 (16)
Overweight	1174 (31)	2018 (31)
Underweight	290 (7.8)	555 (8.4)
Smoking status, No. (%)		
Never	480 (13)	741 (11)
Prior	1617 (43)	1993 (30)
Current	1464 (39)	3088 (47)
Unknown	178 (4.8)	763 (12)
Immunomodulatory drug score, No. (%)		
Good	1585 (42)	1738 (26)
Intermediate	1553 (42)	3123 (47)
Poor	601 (16)	1724 (26)
CCI (excluding malignancies), No. (%)		
0	687 (18)	1429 (22)
1	1142 (31)	2354 (36)
2 to 3	1190 (32)	2017 (31)
4 to 6	552 (15)	679 (10)
7 or higher	168 (4.5)	106 (1.6)
Chronic kidney disease stage, No. (%)		
1	1801 (48)	3592 (55)
2	1350 (36)	2173 (33)
3a	387 (10)	564 (8.6)
3b	147 (3.9)	199 (3.0)
4	41 (1.1)	44 (0.7)
5	13 (0.3)	13 (0.2)
COPD severity, No. (%)		
No COPD	2248 (60)	4225 (64)
COPD, no exacerbations	933 (25)	1564 (24)
COPD, 1–2 exacerbations	459 (12)	687 (10)
COPD, 3 or more exacerbations	99 (2.6)	109 (1.7)
Baseline hypothyroidism	347 (9.3)	391 (5.9)
Baseline autoimmune disease (any)	212 (5.7)	444 (6.7)
ED visit in prior year	2483 (66)	3272 (50)
No. of oncology visits in prior year	3 (2, 6)	3 (1, 6)
Mental health visit in prior year	1225 (33)	1791 (27)
Social work visit in prior year	1457 (39)	1986 (30)
Histology, No. (%)		
Adenocarcinoma	2325 (62)	2976 (45)
Squamous cell	1004 (27)	1762 (27)
NSCLC, NOS	227 (6.1)	1399 (21)
Other	183 (4.9)	448 (6.8)
Tumor PD‐L1 expression, No. (%)		
<1%	656 (18)	0 (0)
1%–49%	843 (23)	0 (0)
50%–100%	1124 (30)	0 (0)
Unknown	1116 (30)	6585 (100)
Immunotherapy regimen type, No. (%)		
Combination with chemotherapy	1685 (45)	0 (NA)
Monotherapy	2054 (55)	0 (NA)
Prior radiotherapy	1436 (38)	2727 (41)
Prior lines of systemic therapy, No. (%)		
0	2669 (71)	4608 (70)
1	1070 (29)	1977 (30)
Time from original diagnosis to treatment initiation (months), No. (%)		
0–6	2978 (80)	5351 (81)
7–12	437 (12)	837 (13)
13–18	161 (4.3)	230 (3.5)
19–24	63 (1.7)	79 (1.2)
24 or more	100 (2.7)	88 (1.3)

Abbreviations: BMI, body mass index; CCI, Charlson Comorbidity Index; COPD, chronic obstructive pulmonary disease; ED, emergency department; NA, not applicable; NSCLC, NOS, non–small cell lung cancer, not otherwise specified; PD‐L1, programmed death ligand‐1.

^a^ No. (%); median (Q1, Q3).

The prevalence of medication users in each cohort (ICI and chemotherapy) are shown in Table S3. The most common medications in the ICI group were opioid analgesics (53%), statins (43%), PPIs (31%), and β‐blockers (30%). The control cohort had similar rates of most medications, although with notably higher use rates of use of opioids and H2As than the ICI cohort (opioids: 72% in control vs. 53% in ICI group; H2As: 43% in control vs. 14% in ICI group).

### Adjusted analyses

The propensity weighting procedure achieved excellent covariate balance in all analyses (Table S4). Table 2 presents the results of the propensity‐weighted regression analyses for each medication class’s effect on OS; Table 3 presents the same analyses for TTNT. After propensity weighting, in the ICI group we did not observe any associations for 15 of 20 medication classes with respect to OS, and for 14 of 20 medication classes with respect to TTNT. For OS, loop diuretics (aHR, 1.25; 95% confidence interval [CI], 1.10–1.43, *p* < .001), anticoagulants (aHR, 1.14; 95% CI, 1.04–1.26, *p* = .0083), opioid analgesics (aHR, 1.28; 95% CI, 1.18–1.38, *p* < .001), penicillin antibiotics (aHR, 1.17; 95% CI, 1.05–1.30, *p* = .0042), and fluoroquinolone antibiotics (aHR, 1.13; 95% CI, 1.00–1.28, *p* = .05) were associated with worse OS. However, when testing these medications in the control group, similar associations in both directionality, magnitude, and statistical significance were observed for each (Table 2). These medications were also associated with inferior TTNT in the ICI group, but the same associations were again observed in the control group, with the exception of a small protective effect of statins in the ICI group that was not replicated in the control group (Table 3). These results were similar in a sensitivity analysis using a 12‐month window for baseline medication use instead of the 3‐month window in the primary analysis (Tables S5 and S6), and when restricting the chemotherapy control group to the years 2010–2015 to increase comparability to the more recently treated ICI group (Table S7 and S8).

**TABLE 2:** Effect of common medications on overall survival.

	ICI cohort		Chemotherapy cohort	
Medication	aHR (95% CI)	*p* ^a^	aHR (95% CI)	*p* ^a^
Statins	0.93 (0.86–1.00)	.059	—^b^	—
PPIs	0.98 (0.91–1.06)	.64	—	—
SSRIs	1.03 (0.92–1.15)	.64	—	—
NSAIDs	1.04 (0.95–1.14)	.4	—	—
H2As	1.09 (0.98–1.21)	.12	—	—
SNRIs	1.06 (0.90–1.25)	.47	—	—
Antipsychotics	1.08 (0.92–1.26)	.34	—	—
β‐Blockers	1.08 (0.99–1.17)	.073	—	—
ACE‐I/ARBs	0.95 (0.88–1.04)	.25	—	—
Antiplatelet	1.01 (0.87–1.19)	.85	—	—
CCBs	1.00 (0.91–1.09)	.99	—	—
Loop diuretics	1.25 (1.10–1.43)	<.001	1.12 (1.02–1.22)	.012
Metformin	1.05 (0.93–1.18)	.4	—	—
Anticoagulants	1.14 (1.04–1.26)	.0083	1.13 (1.04–1.23)	.0039
Opioids	1.28 (1.18–1.38)	<.001	1.20 (1.13–1.27)	<.001
Aspirin	1.00 (0.90–1.12)	.94	—	—
Cephalosporins	1.04 (0.95–1.14)	.43	—	—
Penicillins	1.17 (1.05–1.30)	.0042	1.15 (1.07–1.24)	<.001
Fluoroquinolones	1.13 (1.00–1.28)	.05	1.15 (1.08–1.23)	<.001
Other antibiotic classes	1.03 (0.89–1.20)	.65	—	—

Abbreviations: ACE‐I, angiotensin‐converting enzyme inhibitor; aHR, adjusted hazard ratio; ARB, angiotensin receptor blocker; CCB, calcium channel blocker; CI, confidence interval; H2A, histamine‐2 receptor antagonist; ICI, immune checkpoint inhibitors; NSAID, nonsteroidal anti‐inflammatory drug; PPI, proton‐pump inhibitor; SNRI, serotonin‐norepinephrine reuptake inhibitor; SSRI, selective‐serotonin‐reuptake inhibitor.

^a^ *p* values presented here are nominal (unadjusted for multiple comparisons).

^b^ Analyses not performed due to lack of nominal significance (*p* < .05) in the ICI group.

**TABLE 3:** Effect of common medications on time‐to‐next treatment.

	ICI cohort		Chemotherapy cohort	
Medication	aHR (95% CI)	*p* ^a^	aHR (95% CI)	*p* ^a^
Statins	0.91 (0.84–0.98)	.018	1.00 (0.94–1.06)	.95
PPIs	0.97 (0.90–1.05)	.48	—^b^	—
SSRIs	1.02 (0.92–1.15)	.67	—	—
NSAIDs	1.05 (0.96–1.15)	.26	—	—
H2As	1.05 (0.94–1.16)	.39	—	—
SNRIs	0.99 (0.85–1.17)	.94	—	—
Antipsychotics	1.05 (0.90–1.21)	.55	—	—
β‐Blockers	1.03 (0.95–1.12)	.50	—	—
ACE‐I/ARBs	0.93 (0.86–1.01)	.097	—	—
Antiplatelet	1.01 (0.87–1.19)	.86	—	—
CCBs	0.97 (0.89–1.06)	.55	—	—
Loop diuretics	1.17 (1.03–1.33)	.017	1.15 (1.05–1.25)	.0015
Metformin	1.06 (0.95–1.20)	.29	—	—
Anticoagulants	1.11 (1.01–1.22)	.035	1.12 (1.03–1.22)	.0082
Opioids	1.23 (1.14–1.32)	<.001	1.15 (1.08–1.21)	<.001
Aspirin	0.97 (0.87–1.07)	.52	—	—
Cephalosporins	1.04 (0.95–1.14)	.38	—	—
Penicillins	1.16 (1.04–1.28)	.0055	1.14 (1.06–1.22)	<.001
Fluoroquinolones	1.20 (1.06–1.35)	.0035	1.15 (1.08–1.23)	<.001
Other antibiotic classes	0.97 (0.85–1.12)	.69	—	—

Abbreviations: ACE‐I, angiotensin‐converting enzyme inhibitor; aHR, adjusted hazard ratio; ARB, angiotensin receptor blocker; CCB, calcium channel blocker; CI, confidence interval; H2A, histamine‐2 receptor antagonist; ICI, immune checkpoint inhibitors; NSAID, nonsteroidal anti‐inflammatory drug; PPI, proton‐pump inhibitor; SNRI, serotonin‐norepinephrine reuptake inhibitor; SSRI, selective‐serotonin‐reuptake inhibitor.

^a^ *p* values presented here are nominal (unadjusted for multiple comparisons).

^b^ Analyses not performed due to lack of nominal significance (*p* < .05) in the ICI group.

### Impact of the immunomodulatory drug score on ICI efficacy

**FIGURE 1:**
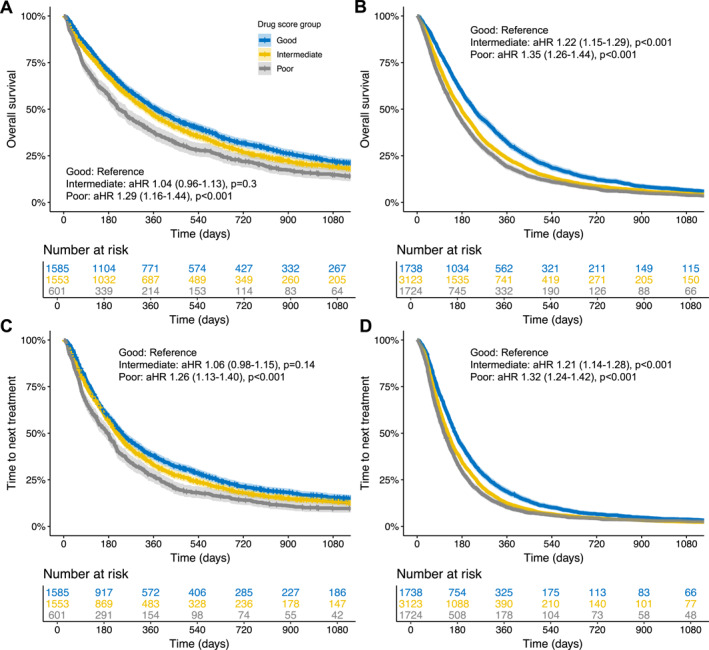
Impact of the immunomodulatory drug score on ICI efficacy. Impact of the immunomodulatory drug score on overall survival (top row) or time to next treatment (bottom row) among patients with metastatic non–small cell lung cancer treated with first or second line ICI (A and C) or chemotherapy (B and D). aHR indicates adjusted hazard ratio; ICI, immune checkpoint inhibitors.

We observed that the immunomodulatory drug score (composite of pre‐baseline antibiotic, corticosteroid, and PPI use) was associated with inferior OS (Figure 1A) and TTNT (Figure 1C) among ICI patients. This association remained robust in multivariable Cox regression (OS: aHR, 1.29 for poor vs. good drug score; 95% CI, 1.16–1.44, *p* < .001; TTNT: aHR 1.26, for poor vs. good drug score; 95% CI, 1.13–1.40, *p* < .001) (Tables S9 and S10). However, similar results were found in the control group (OS: aHR, 1.35 for poor vs. good score; 95% CI, 1.26–1.44, *p* < .001; TTNT: aHR, 1.32 for poor vs. good score; 95% CI, 1.24–1.42, *p* < .001) (Figure 1B,D).

## DISCUSSION

Our retrospective cohort study of veterans with de novo metastatic NSCLC found no evidence that commonly prescribed chronic medications affect oncologic outcomes in patients treated with ICIs. Although loop diuretics, anticoagulants, opioids, penicillin antibiotics, and fluoroquinolone antibiotics were initially associated with worse OS and TTNT in the ICI cohort, nearly identical associations were observed in a chemotherapy‐treated control group. This strongly suggests that these findings are driven by unmeasured confounding rather than true drug–ICI interactions. These results were consistent across sensitivity analyses and are clinically plausible, as patients with volume overload, infection, thrombotic events, or significant pain at diagnosis are likely to have poorer outcomes regardless of treatment type. Similarly, a previously published immunomodulatory drug score—based on recent use of PPIs, corticosteroids, and antibiotics—was associated with worse outcomes in the ICI cohort but showed the same association in chemotherapy‐treated patients, indicating it is a general prognostic marker rather than a modifier of ICI efficacy. Overall, our findings suggest that commonly used medications do not meaningfully influence ICI treatment outcomes in stage IV NSCLC, and prior reports of such associations likely reflect type I error and/or residual confounding.

Our findings contrast with a substantial body of literature suggesting that concomitant medications can modulate ICI efficacy. Although many of these studies are small, single‐institution cohorts susceptible to type I error, several meta‐analyses have reported seemingly robust associations between ICI outcomes and use of medications such as PPIs,^34^ statins,^9^ and antibiotics.^35^ However, the studies included in these meta‐analyses often lacked adequate control for confounding and did not include negative control groups. The absence of negative controls is a pervasive limitation in this literature and significantly undermines causal inference. In observational data sets, medication use frequently reflects underlying illness severity or clinical context—factors that are rarely fully captured. As a result, modest associations may simply reflect unmeasured prognostic differences rather than true drug–ICI interactions.

Among dozens of prior studies, we identified only two that included a chemotherapy‐treated control group. Both were based on the same multi‐institutional data set of 950 ICI‐treated NSCLC patients with PD‐L1 ≥50% and 595 chemotherapy‐treated controls.^28^, ^29^ Although those analyses reported selective effects of antibiotics, corticosteroids, and PPIs in the ICI group, their interpretability is limited by relatively small sample sizes, overlapping patient populations, limited confounding control, and the absence of an external validation cohort. Although an immunomodulatory drug score derived from this cohort was later validated as a prognostic tool in an independent data set,^31^ that validation did not include a chemotherapy control group, leaving open the question of ICI specificity.

In contrast, our study in a diverse national health system demonstrates that this immunomodulatory drug score is associated with poorer outcomes in both ICI‐ and chemotherapy‐treated patients, indicating that it is likely a general prognostic marker rather than a specific modulator of ICI efficacy. Likewise, the associations we observed between worse survival and use of loop diuretics, antibiotics, anticoagulants, and opioids were similarly present in the chemotherapy cohort, reinforcing the interpretation that these drugs reflect baseline prognosis rather than direct interaction with immunotherapy. Together, these findings underscore the importance of rigorous observational study design—particularly the use of large, diverse cohorts, robust confounding adjustment, and inclusion of appropriate controls. These elements are essential to distinguishing true drug–ICI interactions from spurious associations and enhancing the reliability of real‐world evidence in immuno‐oncology.

Our study has several limitations. First, as an observational analysis, we cannot entirely exclude residual confounding by factors we could not measure. For example, we lacked detailed data on patients’ diet, nutritional status, or gut microbiome composition—all potential modulators of ICI response that might correlate with medication use.^36^ Second, our cohort was drawn from the VHA and thus consists predominantly of older male patients; as such, the generalizability to younger populations or female patients is limited. The external validity of our findings should be confirmed in other health care settings. Third, we defined concomitant medication exposure based on prescription and timing relative to ICI initiation, but misclassification is possible (e.g., over‐the‐counter PPIs not captured, or patients taking medications irregularly). Fourth, although our inclusion of a chemotherapy‐treated control group is a major strength, we acknowledge that patients selected for ICI versus chemotherapy might differ in ways not fully corrected by propensity weighting. In addition, we recognize that advancements in supportive care and evolving medication indications across our study period could have produced shifts in the causal structure of confounding over time, which could undermine our results. To mitigate this, we performed a sensitivity analysis restricting the chemotherapy control group to a more modern period (2010–2015), which produced similar results to the primary analysis. Fifth, to build a homogeneous sample, we included only patients with NSCLC and therefore did not assess other common solid malignancies, such as melanoma, in which outcomes are drastically improved with ICI therapy and an interaction effect may be more clinically significant or easily detectable. Finally, we did not assess objective response rate or radiographic progression‐free survival.

In summary, our findings do not support the notion that use of antibiotics, PPIs, H2 antagonists, opioids, or other common supportive medications meaningfully compromises the efficacy of immune checkpoint inhibitors in NSCLC. By leveraging a large national cohort with a chemotherapy‐treated control group, we provide evidence that any medication‐specific modulation of ICI efficacy is, at most, modest and potentially noncausal. These data provide reassurance that optimal cancer care should continue to prioritize evidence‐based management of comorbid conditions alongside immunotherapy.

## AUTHOR CONTRIBUTIONS

**Daria Brinzevich**: Conceptualization; investigation; writing—original draft; writing—review and editing. **Virginia Falvello**: Conceptualization; investigation; writing—original draft; writing—review and editing. **Sadiq S. Rehmani**: Conceptualization; investigation; writing—original draft; writing—review and editing. **Garth W. Strohbehn**: Conceptualization; methodology; writing—review and editing. **Nithya Ramnath**: Conceptualization; writing—review and editing. **Michael P. Dykstra**: Writing—review and editing. **Luke M. Higgins**: Writing—review and editing. **David Elliott**: Resources; writing—review and editing. **Matthew J. Schipper**: Methodology; writing—review and editing. **Michael D. Green**: Conceptualization; methodology; writing—review and editing. **Alex K. Bryant**: Conceptualization; methodology; formal analysis; supervision; project administration; writing—original draft; writing—review and editing.

## CONFLICT OF INTEREST STATEMENT

Luke M. Higgins reports travel funding from the American Society for Radiation Oncology. Matthew J. Schipper reports consulting fees from Innovative Analytics. Garth W. Strohbehn reports consulting fees from EBSCO Industries Inc and VIVIO Health; and participation as a fiduciary officer for Optimal Cancer Care Alliance. The other authors declare no conflicts of interest.

## Supporting information

Supplementary Material

## Data Availability

Data is not available for sharing at this time.
