# Supplementary material for: The effect of common medications on the efficacy of immune checkpoint inhibitors
Source: Cancer. 2025 Dec 14;131(24):e70222. doi: 10.1002/cncr.70222 (PMC12701955; doi:10.1002/cncr.70222)
Supplement: Supplementary file 1 — Supplementary Material [file CNCR-131-e70222-s001.docx]

**Supplemental Material for:**

**The effect of common medications on the efficacy of immune checkpoint inhibitors.**

Daria Brinzevich^1^, Virginia Falvello^1^, Sadiq Rehmani^1^, Garth Strohbehn^1,2,3,4,5,6^, Nithya Ramnath^1,3,5,6^, Michael Dykstra^7,8^, Luke M. Higgins,^8^ David Elliott^7,8^, Matthew Schipper,^7,9^ Michael D. Green^10^, and Alex K. Bryant^2,4,6,7,8^

^1^Division of Hematology/Oncology, Department of Medicine, University of Michigan, Ann Arbor, MI

^2^Veterans Affairs Center for Clinical Management Research, Ann Arbor, MI

^3^Division of Oncology, LTC Charles S. Kettles VA Medical Center, Ann Arbor, MI

^4^Institute for Health Policy and Innovation, University of Michigan, Ann Arbor, MI

^5^Lung Precision Oncology Program, Charles S. Kettles VA Medical Center, Ann Arbor, MI

^6^Rogel Cancer Center, Michigan Medicine, Ann Arbor, MI

^7^Department of Radiation Oncology, Veterans Affairs Ann Arbor Healthcare System, Ann Arbor MI

^8^Department of Radiation Oncology, University of Michigan, Ann Arbor MI

^9^Department of Biostatistics, University of Michigan, Ann Arbor, MI

^10^Anchorage Radiation Oncology Center, Anchorage AK

**Page 2**: Supplemental Table 1. Systemic therapy regimens included in the immunotherapy and chemotherapy cohorts.

**Page 3**: Supplemental Table 2. Specific drugs comprising medication classes in the primary analysis.

**Page 4:** Supplemental Table 3. Prevalence of medication use in ICI and chemotherapy cohorts.

**Page 5:** Supplemental Table 4. Proportion of variables in each analysis with good (standardized difference <0.10) or excellent (<0.05) balance after propensity weighting.

**Page 6**: Supplemental Table 5. Sensitivity analysis for overall survival using a 12-month window prior to treatment to define medication use.

**Page 7**: Supplemental Table 6. Sensitivity analysis for time to next treatment using a 12-month window prior to treatment to define medication use.

**Page 8:** Supplemental Table 7. Sensitivity analysis for overall survival restricting chemotherapy cohort to years 2010-2015.

**Page 9:** Supplemental Table 8. Sensitivity analysis for time to next treatment restricting chemotherapy cohort to years 2010-2015.

**Page 10**: Supplemental Table 9. Results of the multivariable Cox regression for overall survival.

**Page 12**: Supplemental Table 10. Results of the multivariable Cox regression for time to next treatment.

**Supplemental Table 1.** Systemic therapy regimens included in the immunotherapy and chemotherapy cohorts.

| **Cohort** | **Regimen** | **n** |
| --- | --- | --- |
| Immunotherapy | pembrolizumab | 1,320 |
| Immunotherapy | carboplatin/pembrolizumab/pemetrexed | 1,158 |
| Immunotherapy | nivolumab | 734 |
| Immunotherapy | carboplatin/paclitaxel/pembrolizumab | 527 |
| Chemotherapy | carboplatin/paclitaxel | 2,278 |
| Chemotherapy | pemetrexed | 819 |
| Chemotherapy | carboplatin/pemetrexed | 723 |
| Chemotherapy | docetaxel | 602 |
| Chemotherapy | carboplatin/gemcitabine | 474 |
| Chemotherapy | bevacizumab/carboplatin/paclitaxel | 321 |
| Chemotherapy | carboplatin/docetaxel | 291 |
| Chemotherapy | gemcitabine | 261 |
| Chemotherapy | cisplatin/pemetrexed | 158 |
| Chemotherapy | cisplatin/etoposide | 147 |
| Chemotherapy | vinorelbine | 122 |
| Chemotherapy | carboplatin/etoposide | 89 |
| Chemotherapy | bevacizumab/carboplatin/pemetrexed | 86 |
| Chemotherapy | cisplatin/gemcitabine | 83 |
| Chemotherapy | paclitaxel | 68 |
| Chemotherapy | cisplatin/docetaxel | 63 |

**Supplemental Table 2**. Specific drugs comprising medication classes in the primary analysis.

| **Drug Class** | **Drug** |
| --- | --- |
| Beta blockers | Metoprolol, carvedilol, atenolol, bisoprolol, nadolol, propranolol, labetalol, sotalol |
| Calcium channel blockers | Amlodipine, nifedipine, diltiazem, verapamil |
| ACE-I/ARBs | Lisinopril, benzapril, enalapril, losartan, valsartan, olmesartan, telmisartan |
| Loop diuretics | Furosemide, torsemide, bumetanide |
| Statins | Atorvastatin, rosuvastatin, simvastatin, pravastatin, lovastatin |
| Metformin | Metformin |
| PPI | Omeprazole, esomeprazole, pantoprazole, lansoprazole, dexlansoprazole, rabeprazole |
| H2As | Famotidine, ranitidine, cimetidine |
| SSRIs | Sertraline, citalopram, escitalopram, fluoxetine, fluvoxamine, paroxetine, vortioxetine, dapoxetine |
| SNRIs | Venlafaxine, duloxetine |
| Anti-psychotics | Risperidone, olanzapine, quetiapine, aripiprazole, ziprasidone, lurasidone, clozapine, haloperidol |
| NSAIDs | Ibuprofen, naproxen, diclofenac, celecoxib, meloxicam, ketorolac, sulindac |
| Aspirin | Aspirin |
| Opioids | Oxycodone, fentanyl, codeine, hydrocodone, morphine, mthadone, buprenorphine, hydromorphone, tramadol, meperidine |
| Anti-platelets | Clopidogrel, ticagrelor, prasugrel, cilostazol, dipyridamole |
| Anti-coagulants | Warfarin, apixaban, dabigatran, edoxaban, rivaroxaban, fondaparinux, enoxaparin |

Abbreviations: PPI: proton-pump inhibitor, SSRI: selective-serotonin-reuptake inhibitor, NSAID: non-steroidal anti-inflammatory drug, H2A: histamine-2 receptor antagonist, SNRI: serotonin-norepinephrine reuptake inhibitor, ACE-I: angiotensin-converting enzyme inhibitor , ARB: angiotensin receptor blocker, CCB: calcium channel blocker

Specific antibiotic classes included all individual antibiotics of each type (cephalosporins, penicillins, fluoroquinolones, or other antibiotics not included in these three specific classes).

**Supplemental Table 3.** Prevalence of medication use in ICI and chemotherapy cohorts.

| **Characteristic** | **Immunotherapy**  N = 3,739^1^ | **Chemotherapy**  N = 6,585^1^ |
| --- | --- | --- |
| **Opioids** | 1,985 (53%) | 4,715 (72%) |
| **Statins** | 1,608 (43%) | 2,178 (33%) |
| **PPIs** | 1,145 (31%) | 2,492 (38%) |
| **Beta Blockers** | 1,119 (30%) | 1,932 (29%) |
| **ACE-I / ARBs** | 1,101 (29%) | 1,886 (29%) |
| **CCBs** | 850 (23%) | 1,166 (18%) |
| **Cephalosporins** | 848 (23%) | 1,366 (21%) |
| **Anticoagulants** | 604 (16%) | 644 (9.8%) |
| **Penicillins** | 550 (15%) | 960 (15%) |
| **Aspirin** | 510 (14%) | 914 (14%) |
| **H2As** | 509 (14%) | 2,857 (43%) |
| **Metformin** | 470 (13%) | 554 (8.4%) |
| **SSRIs** | 425 (11%) | 890 (14%) |
| **Fluoroquinolones** | 346 (9.3%) | 1,456 (22%) |
| **Loop diuretics** | 343 (9.2%) | 678 (10%) |
| **Antipsychotics** | 231 (6.2%) | 311 (4.7%) |
| **Other antibiotic classes** | 240 (6.4%) | 466 (7.1%) |
| **Anti-platelets** | 213 (5.7%) | 289 (4.4%) |
| **SNRIs** | 199 (5.3%) | 87 (1.3%) |
| ^1^n (%) | | |

Abbreviations: PPI: proton-pump inhibitor, SSRI: selective-serotonin-reuptake inhibitor, NSAID: non-steroidal anti-inflammatory drug, H2A: histamine-2 receptor antagonist, SNRI: serotonin-norepinephrine reuptake inhibitor, ACE-I: angiotensin-converting enzyme inhibitor , ARB: angiotensin receptor blocker, CCB: calcium channel blocker; aHR: adjusted hazard ratio; ICI: immune checkpoint inhibitors.

**Supplemental Table 4.** Proportion of variables in each analysis with good (standardized mean difference <0.10) or excellent (<0.05) balance after propensity weighting.

| **Analysis** | **Immunotherapy**  N = 3,739^1^ | | **Chemotherapy**  N = 6,585^1^ | |
| --- | --- | --- | --- | --- |
|  | **SD <0.05** | **SD <0.10** | **SD <0.05** | **SD <0.10** |
| Statins | 0.98 | 1.00 | 1.00 | 1.00 |
| PPIs | 0.98 | 1.00 | --^1^ | -- |
| SSRIs | 0.95 | 1.00 | -- | -- |
| NSAIDs | 0.96 | 1.00 | -- | -- |
| H2As | 0.96 | 1.00 | -- | -- |
| SNRIs | 0.84 | 1.00 | -- | -- |
| Antipsychotics | 0.81 | 1.00 | -- | -- |
| Beta Blockers | 0.95 | 1.00 | -- | -- |
| ACE-I / ARBs | 1.00 | 1.00 | -- | -- |
| Anti-platelet | 0.96 | 1.00 | -- | -- |
| CCBs | 0.98 | 1.00 | -- | -- |
| Loop diuretics | 0.75 | 0.95 | 0.96 | 1.00 |
| Metformin | 0.95 | 1.00 | -- | -- |
| Anticoagulants | 0.93 | 1.00 | 1.00 | 1.00 |
| Opioids | 0.96 | 1.00 | 0.98 | 1.00 |
| Aspirin | 1.00 | 1.00 | -- | -- |
| Cephalosporins | 0.96 | 1.00 | -- | -- |
| Penicillins | 0.93 | 1.00 | 1.00 | 1.00 |
| Fluoroquinolones | 0.91 | 1.00 | 0.98 | 1.00 |
| Other antibiotic classes | 0.95 | 0.96 | -- | -- |

Abbreviations: PPI: proton-pump inhibitor, SSRI: selective-serotonin-reuptake inhibitor, NSAID: non-steroidal anti-inflammatory drug, H2A: histamine-2 receptor antagonist, SNRI: serotonin-norepinephrine reuptake inhibitor, ACE-I: angiotensin-converting enzyme inhibitor , ARB: angiotensin receptor blocker, CCB: calcium channel blocker; aHR: adjusted hazard ratio; ICI: immune checkpoint inhibitors.

^1^Analyses not performed due to lack of nominal significance (p<0.05) in the ICI group.

**Supplemental Table 5.** Sensitivity analysis for overall survival using a 12-month window prior to treatment to define medication use.

|  | **ICI Cohort** | | **Chemotherapy cohort** | |
| --- | --- | --- | --- | --- |
| **Medication** | **aHR (95% CI)** | **p-value**^1^ | **aHR (95% CI)** | **p-value**^1^ |
| Statins | 0.90 (0.83-0.98) | **0.015** | 1.02 (0.97-1.08) | 0.4 |
| PPIs | 0.98 (0.91-1.06) | 0.61 | --^2^ | -- |
| SSRIs | 1.02 (0.91-1.13) | 0.78 | -- | -- |
| NSAIDs | 1.01 (0.94-1.10) | 0.72 | -- | -- |
| H2As | 1.02 (0.93-1.12) | 0.65 | -- | -- |
| SNRIs | 1.11 (0.97-1.28) | 0.13 | -- | -- |
| Antipsychotics | 0.98 (0.85-1.14) | 0.81 | -- | -- |
| Beta Blockers | 1.11 (1.03-1.21) | **0.0087** | 0.97 (0.92-1.02) | 0.29 |
| ACE-I / ARBs | 1.02 (0.94-1.11) | 0.67 | -- | -- |
| Anti-platelet | 0.98 (0.86-1.11) | 0.73 | -- | -- |
| CCBs | 1.02 (0.94-1.10) | 0.7 | -- | -- |
| Loop diuretics | 1.23 (1.09-1.37) | **<0.001** | 1.07 (0.99-1.16) | 0.067 |
| Metformin | 1.05 (0.94-1.17) | 0.39 | -- | -- |
| Anticoagulants | 1.14 (1.04-1.25) | **0.0069** | 1.08 (1.00-1.17) | **0.044** |
| Opioids | 1.22 (1.12-1.32) | **<0.001** | 1.12 (1.05-1.19) | **<0.001** |
| Aspirin | 1.03 (0.94-1.13) | 0.52 | -- | -- |
| Cephalosporins | 1.03 (0.95-1.12) | 0.45 | -- | -- |
| Penicillins | 1.10 (1.01-1.20) | **0.033** | 1.06 (1.00-1.12) | **0.049** |
| Fluoroquinolones | 1.04 (0.95-1.15) | 0.42 | -- | -- |
| Other antibiotic classes | 1.03 (0.90-1.18) | 0.65 | -- | -- |

Abbreviations: PPI: proton-pump inhibitor, SSRI: selective-serotonin-reuptake inhibitor, NSAID: non-steroidal anti-inflammatory drug, H2A: histamine-2 receptor antagonist, SNRI: serotonin-norepinephrine reuptake inhibitor, ACE-I: angiotensin-converting enzyme inhibitor , ARB: angiotensin receptor blocker, CCB: calcium channel blocker; aHR: adjusted hazard ratio; ICI: immune checkpoint inhibitors.

Bolded results indicated nominal significance (p<0.05).

^1^P values presented here are nominal (unadjusted for multiple comparisons).

^2^Analyses not performed due to lack of nominal significance (p<0.05) in the ICI group.

**Supplemental Table 6.** Sensitivity analysis for time to next treatment using a 12-month window prior to treatment to define medication use.

|  | **ICI Cohort** | | **Chemotherapy cohort** | |
| --- | --- | --- | --- | --- |
| **Medication** | **aHR (95% CI)** | **p-value**^1^ | **aHR (95% CI)** | **p-value**^1^ |
| Statins | 0.91 (0.84-0.98) | **0.015** | 1.00 (0.95-1.06) | 0.9 |
| PPIs | 0.98 (0.91-1.05) | 0.56 | --^2^ | -- |
| SSRIs | 0.97 (0.88-1.08) | 0.56 | -- | -- |
| NSAIDs | 1.03 (0.96-1.12) | 0.4 | -- | -- |
| H2As | 1.02 (0.93-1.11) | 0.74 | -- | -- |
| SNRIs | 1.06 (0.93-1.22) | 0.4 | -- | -- |
| Antipsychotics | 0.94 (0.82-1.08) | 0.38 | -- | -- |
| Beta Blockers | 1.06 (0.98-1.15) | 0.14 | -- | -- |
| ACE-I / ARBs | 0.99 (0.92-1.08) | 0.88 | -- | -- |
| Anti-platelet | 0.99 (0.87-1.12) | 0.88 | -- | -- |
| CCBs | 0.98 (0.90-1.06) | 0.55 | -- | -- |
| Loop diuretics | 1.15 (1.03-1.28) | **0.016** | 1.06 (0.99-1.15) | 0.11 |
| Metformin | 1.06 (0.96-1.18) | 0.24 | -- | -- |
| Anticoagulants | 1.09 (1.00-1.20) | 0.061 | -- | -- |
| Opioids | 1.19 (1.10-1.29) | **<0.001** | 1.09 (1.02-1.16) | **0.012** |
| Aspirin | 0.99 (0.90-1.08) | 0.79 | -- | -- |
| Cephalosporins | 1.03 (0.95-1.11) | 0.52 | -- | -- |
| Penicillins | 1.08 (0.99-1.17) | 0.083 | -- | -- |
| Fluoroquinolones | 1.06 (0.96-1.16) | 0.24 | -- | -- |
| Other antibiotic classes | 0.99 (0.87-1.12) | 0.84 | -- | -- |

Abbreviations: PPI: proton-pump inhibitor, SSRI: selective-serotonin-reuptake inhibitor, NSAID: non-steroidal anti-inflammatory drug, H2A: histamine-2 receptor antagonist, SNRI: serotonin-norepinephrine reuptake inhibitor, ACE-I: angiotensin-converting enzyme inhibitor , ARB: angiotensin receptor blocker, CCB: calcium channel blocker; aHR: adjusted hazard ratio; ICI: immune checkpoint inhibitors.

Bolded results indicated nominal significance (p<0.05).

^1^P values presented here are nominal (unadjusted for multiple comparisons).

^2^Analyses not performed due to lack of nominal significance (p<0.05) in the ICI group.

**Supplemental Table 7.** Sensitivity analysis for overall survival restricting chemotherapy cohort to years 2010-2015.

|  | **ICI Cohort** | | **Chemotherapy cohort** | |
| --- | --- | --- | --- | --- |
| **Medication** | **aHR (95% CI)** | **p-value**^1^ | **aHR (95% CI)** | **p-value**^1^ |
| Statins | 0.93 (0.86-1.00) | 0.059 | --^2^ | -- |
| PPIs | 0.98 (0.91-1.06) | 0.64 | -- | -- |
| SSRIs | 1.03 (0.92-1.15) | 0.64 | -- | -- |
| NSAIDs | 1.04 (0.95-1.14) | 0.4 | -- | -- |
| H2As | 1.09 (0.98-1.21) | 0.12 | -- | -- |
| SNRIs | 1.06 (0.90-1.25) | 0.47 | -- | -- |
| Antipsychotics | 1.08 (0.92-1.26) | 0.34 | -- | -- |
| Beta Blockers | 1.08 (0.99-1.17) | 0.073 | -- | -- |
| ACE-I / ARBs | 0.95 (0.88-1.04) | 0.25 | -- | -- |
| Anti-platelet | 1.01 (0.87-1.19) | 0.85 | -- | -- |
| CCBs | 1.00 (0.91-1.09) | 1.00 | -- | -- |
| Loop diuretics | 1.25 (1.10-1.43) | **<0.001** | 1.10 (0.98-1.23) | 0.11 |
| Metformin | 1.05 (0.93-1.18) | 0.4 | -- | **--** |
| Anticoagulants | 1.14 (1.04-1.26) | **0.0083** | 1.16 (1.04-1.29) | **0.0085** |
| Opioids | 1.28 (1.18-1.38) | **<0.001** | 1.16 (1.07-1.25) | **<0.001** |
| Aspirin | 1.00 (0.90-1.12) | 0.94 | -- | -- |
| Cephalosporins | 1.04 (0.95-1.14) | 0.43 | -- | -- |
| Penicillins | 1.17 (1.05-1.30) | **0.0042** | 1.14 (1.03-1.25) | **0.0092** |
| Fluoroquinolones | 1.13 (1.00-1.28) | **0.05** | 1.12 (1.04-1.22) | **0.0053** |
| Other antibiotic classes | 1.03 (0.89-1.20) | 0.65 | -- | -- |

Abbreviations: PPI: proton-pump inhibitor, SSRI: selective-serotonin-reuptake inhibitor, NSAID: non-steroidal anti-inflammatory drug, H2A: histamine-2 receptor antagonist, SNRI: serotonin-norepinephrine reuptake inhibitor, ACE-I: angiotensin-converting enzyme inhibitor , ARB: angiotensin receptor blocker, CCB: calcium channel blocker; aHR: adjusted hazard ratio; ICI: immune checkpoint inhibitors.

Bolded results indicated nominal significance (p<0.05).

^1^P values presented here are nominal (unadjusted for multiple comparisons).

^2^Analyses not performed due to lack of nominal significance (p<0.05) in the ICI group.

**Supplemental Table 8. Sensitivity analysis for time to next treatment restricting chemotherapy cohort to years 2010-2015.**

|  | **ICI Cohort** | | **Chemotherapy cohort** | |
| --- | --- | --- | --- | --- |
| **Medication** | **aHR (95% CI)** | **p-value**^1^ | **aHR (95% CI)** | **p-value**^1^ |
| Statins | 0.91 (0.84-0.98) | **0.018** | 1.03 (0.96-1.11) | 0.43 |
| PPIs | 0.97 (0.90-1.05) | 0.48 | --^2^ | -- |
| SSRIs | 1.02 (0.92-1.15) | 0.67 | -- | -- |
| NSAIDs | 1.05 (0.96-1.15) | 0.26 | -- | -- |
| H2As | 1.05 (0.94-1.16) | 0.39 | -- | -- |
| SNRIs | 0.99 (0.85-1.17) | 0.94 | -- | -- |
| Antipsychotics | 1.05 (0.90-1.21) | 0.55 | -- | -- |
| Beta Blockers | 1.03 (0.95-1.12) | 0.5 | -- | -- |
| ACE-I / ARBs | 0.93 (0.86-1.01) | 0.097 | -- | -- |
| Anti-platelet | 1.01 (0.87-1.19) | 0.86 | -- | -- |
| CCBs | 0.97 (0.89-1.06) | 0.55 | -- | -- |
| Loop diuretics | 1.17 (1.03-1.33) | **0.017** | 1.16 (1.04-1.30) | **0.0079** |
| Metformin | 1.06 (0.95-1.20) | 0.29 | -- | **--** |
| Anticoagulants | 1.11 (1.01-1.22) | **0.035** | 1.12 (1.00-1.25) | **0.048** |
| Opioids | 1.23 (1.14-1.32) | **<0.001** | 1.15 (1.06-1.24) | **<0.001** |
| Aspirin | 0.97 (0.87-1.07) | 0.52 | -- | -- |
| Cephalosporins | 1.04 (0.95-1.14) | 0.38 | -- | -- |
| Penicillins | 1.16 (1.04-1.28) | **0.0055** | 1.16 (1.05-1.27) | **0.0029** |
| Fluoroquinolones | 1.20 (1.06-1.35) | **0.0035** | 1.15 (1.06-1.25) | **0.001** |
| Other antibiotic classes | 0.97 (0.85-1.12) | 0.69 | -- | -- |

Abbreviations: PPI: proton-pump inhibitor, SSRI: selective-serotonin-reuptake inhibitor, NSAID: non-steroidal anti-inflammatory drug, H2A: histamine-2 receptor antagonist, SNRI: serotonin-norepinephrine reuptake inhibitor, ACE-I: angiotensin-converting enzyme inhibitor , ARB: angiotensin receptor blocker, CCB: calcium channel blocker; aHR: adjusted hazard ratio; ICI: immune checkpoint inhibitors.

Bolded results indicated nominal significance (p<0.05).

^1^P values presented here are nominal (unadjusted).

^2^Analyses not performed due to lack of nominal significance (p<0.05) in the ICI group.

**Supplemental Table 9**. Results of the multivariable Cox regressions for overall survival.

|  | **Immunotherapy** | | | **Chemotherapy** | | |
| --- | --- | --- | --- | --- | --- | --- |
| **Characteristic** | **HR**^1^ | **95% CI**^1^ | **p-value** | **HR**^1^ | **95% CI**^1^ | **p-value** |
| Immunomodulatory drug score |  |  |  |  |  |  |
| Good | — | — |  | — | — |  |
| Intermediate | 1.04 | 0.96, 1.13 | 0.3 | 1.22 | 1.15, 1.29 | <0.001 |
| Poor | 1.29 | 1.16, 1.44 | <0.001 | 1.35 | 1.26, 1.44 | <0.001 |
| Age group (years) |  |  |  |  |  |  |
| 59 or less | — | — |  | — | — |  |
| 60 to 69 | 1.05 | 0.92, 1.20 | 0.4 | 1.01 | 0.95, 1.07 | 0.8 |
| 70 to 79 | 1.14 | 0.99, 1.31 | 0.061 | 1.08 | 0.99, 1.16 | 0.074 |
| 80 or higher | 1.42 | 1.19, 1.70 | <0.001 | 1.15 | 1.02, 1.30 | 0.018 |
| Marital status |  |  |  |  |  |  |
| Divorced | — | — |  | — | — |  |
| Married | 0.99 | 0.91, 1.08 | 0.8 | 1.00 | 0.94, 1.06 | >0.9 |
| Never married | 1.00 | 0.88, 1.14 | >0.9 | 0.94 | 0.85, 1.03 | 0.2 |
| Other | 1.05 | 0.93, 1.19 | 0.4 | 1.02 | 0.94, 1.11 | 0.6 |
| Sex |  |  |  |  |  |  |
| Female | — | — |  | — | — |  |
| Male | 1.20 | 0.97, 1.50 | 0.094 | 1.34 | 1.15, 1.56 | <0.001 |
| Self-reported race |  |  |  |  |  |  |
| Non-Hispanic White | — | — |  | — | — |  |
| Non-Hispanic Black | 0.81 | 0.74, 0.89 | <0.001 | 0.96 | 0.90, 1.03 | 0.2 |
| Hispanic | 0.94 | 0.76, 1.16 | 0.5 | 0.94 | 0.79, 1.12 | 0.5 |
| Other | 0.92 | 0.69, 1.21 | 0.5 | 0.82 | 0.65, 1.03 | 0.087 |
| Unknown | 1.04 | 0.87, 1.24 | 0.7 | 1.15 | 1.05, 1.26 | 0.002 |
| BMI group |  |  |  |  |  |  |
| Healthy weight | — | — |  | — | — |  |
| Obese | 0.86 | 0.78, 0.96 | 0.005 | 0.82 | 0.76, 0.88 | <0.001 |
| Overweight | 0.96 | 0.88, 1.04 | 0.3 | 0.89 | 0.84, 0.94 | <0.001 |
| Underweight | 1.23 | 1.07, 1.42 | 0.003 | 1.41 | 1.29, 1.55 | <0.001 |
| Histology |  |  |  |  |  |  |
| Adenocarcinoma | — | — |  | — | — |  |
| Squamous cell | 1.24 | 1.14, 1.35 | <0.001 | 1.06 | 1.00, 1.12 | 0.069 |
| NSCLC, NOS | 1.07 | 0.92, 1.24 | 0.4 | 1.04 | 0.98, 1.11 | 0.2 |
| Other | 0.90 | 0.75, 1.07 | 0.2 | 1.08 | 0.98, 1.20 | 0.11 |
| Prior radiotherapy | 1.07 | 0.99, 1.16 | 0.071 | 1.07 | 1.02, 1.13 | 0.006 |
| Prior lines of systemic therapy | 1.02 | 0.92, 1.12 | 0.7 | 0.95 | 0.89, 1.02 | 0.2 |
| Charlson Comorbidity Index (excluding malignancies) |  |  |  |  |  |  |
| 0 | — | — |  | — | — |  |
| 1 | 1.17 | 1.04, 1.31 | 0.007 | 1.02 | 0.95, 1.09 | 0.6 |
| 2 to 3 | 1.19 | 1.06, 1.34 | 0.003 | 1.05 | 0.97, 1.13 | 0.2 |
| 4 to 6 | 1.29 | 1.12, 1.49 | <0.001 | 1.20 | 1.08, 1.33 | <0.001 |
| 7 or higher | 1.45 | 1.19, 1.78 | <0.001 | 1.36 | 1.11, 1.67 | 0.003 |
| Tumor PD-L1 expression |  |  |  |  |  |  |
| <1% | — | — |  | — | — |  |
| 1-49% | 0.93 | 0.83, 1.04 | 0.2 |  |  |  |
| 50-100% | 0.72 | 0.65, 0.81 | <0.001 |  |  |  |
| Unknown | 0.92 | 0.82, 1.02 | 0.13 |  |  |  |
| Smoking status |  |  |  |  |  |  |
| Never | — | — |  | — | — |  |
| Prior | 0.96 | 0.86, 1.08 | 0.5 | 0.98 | 0.90, 1.06 | 0.6 |
| Current | 0.89 | 0.79, 1.00 | 0.052 | 0.96 | 0.88, 1.05 | 0.4 |
| Unknown | 0.98 | 0.81, 1.18 | 0.8 | 0.96 | 0.87, 1.07 | 0.5 |
| COPD severity |  |  |  |  |  |  |
| No COPD | — | — |  | — | — |  |
| COPD, no exacerbations | 0.92 | 0.84, 1.01 | 0.094 | 0.93 | 0.87, 0.99 | 0.020 |
| COPD, 1-2 exacerbations | 1.04 | 0.92, 1.17 | 0.5 | 1.03 | 0.95, 1.12 | 0.5 |
| COPD, 3 or more exacerbations | 1.06 | 0.84, 1.33 | 0.6 | 0.98 | 0.81, 1.20 | 0.9 |
| ED visit in prior year | 1.12 | 1.03, 1.21 | 0.009 | 1.19 | 1.13, 1.25 | <0.001 |
| Social work visit in prior year | 1.14 | 1.06, 1.23 | <0.001 | 1.05 | 1.00, 1.11 | 0.069 |
| Mental health visit in prior year | 0.96 | 0.88, 1.04 | 0.3 | 0.94 | 0.89, 0.99 | 0.028 |
| Chronic kidney disease stage |  |  |  |  |  |  |
| 1 | — | — |  | — | — |  |
| 2 | 0.83 | 0.77, 0.90 | <0.001 | 0.88 | 0.84, 0.94 | <0.001 |
| 3a | 0.92 | 0.81, 1.04 | 0.2 | 0.93 | 0.84, 1.02 | 0.12 |
| 3b | 0.88 | 0.72, 1.07 | 0.2 | 0.92 | 0.79, 1.07 | 0.3 |
| 4 | 0.86 | 0.61, 1.22 | 0.4 | 1.00 | 0.74, 1.35 | >0.9 |
| 5 | 1.79 | 1.00, 3.21 | 0.052 | 0.96 | 0.55, 1.66 | 0.9 |
| Number of oncology visits in prior year | 1.00 | 0.99, 1.01 | 0.6 | 1.00 | 0.99, 1.00 | 0.2 |
| Baseline autoimmune disease (any) | 0.99 | 0.85, 1.16 | >0.9 | 1.05 | 0.95, 1.15 | 0.4 |
| Baseline hypothyroidism | 1.03 | 0.91, 1.16 | 0.7 | 1.01 | 0.91, 1.12 | 0.8 |
| ^1^HR = Hazard Ratio, CI = Confidence Interval | | | | | | |

Abbreviations: COPD: chronic obstructive pulmonary disease; ED: emergency department; NSCLC, NOS: non-small-cell lung cancer, not otherwise specified; PD-L1: programmed death ligand-1.

**Supplemental Table 10**. Results of the multivariable Cox regressions for time to next treatment.

|  | **Immunotherapy** | | | **Chemotherapy** | | |
| --- | --- | --- | --- | --- | --- | --- |
| **Characteristic** | **HR**^1^ | **95% CI**^1^ | **p-value** | **HR**^1^ | **95% CI**^1^ | **p-value** |
| Immunomodulatory drug score |  |  |  |  |  |  |
| Good | — | — |  | — | — |  |
| Intermediate | 1.06 | 0.98, 1.15 | 0.14 | 1.21 | 1.14, 1.28 | <0.001 |
| Poor | 1.26 | 1.13, 1.40 | <0.001 | 1.32 | 1.24, 1.42 | <0.001 |
| Age group (years) |  |  |  |  |  |  |
| 59 or less | — | — |  | — | — |  |
| 60 to 69 | 1.03 | 0.90, 1.17 | 0.7 | 1.00 | 0.94, 1.07 | >0.9 |
| 70 to 79 | 1.10 | 0.97, 1.26 | 0.2 | 1.05 | 0.97, 1.14 | 0.2 |
| 80 or higher | 1.24 | 1.05, 1.47 | 0.014 | 1.06 | 0.95, 1.20 | 0.3 |
| Marital status |  |  |  |  |  |  |
| Divorced | — | — |  | — | — |  |
| Married | 1.02 | 0.94, 1.11 | 0.6 | 1.02 | 0.96, 1.08 | 0.6 |
| Never married | 1.01 | 0.89, 1.14 | 0.9 | 0.94 | 0.85, 1.03 | 0.2 |
| Other | 1.06 | 0.94, 1.20 | 0.3 | 1.00 | 0.92, 1.08 | >0.9 |
| Sex |  |  |  |  |  |  |
| Female | — | — |  | — | — |  |
| Male | 1.08 | 0.88, 1.32 | 0.5 | 1.18 | 1.01, 1.36 | 0.034 |
| Self-reported race |  |  |  |  |  |  |
| Non-Hispanic White | — | — |  | — | — |  |
| Non-Hispanic Black | 0.86 | 0.78, 0.94 | 0.001 | 0.98 | 0.92, 1.05 | 0.6 |
| Hispanic | 0.95 | 0.77, 1.16 | 0.6 | 1.07 | 0.90, 1.27 | 0.5 |
| Other | 1.00 | 0.77, 1.31 | >0.9 | 0.90 | 0.72, 1.13 | 0.4 |
| Unknown | 1.08 | 0.91, 1.28 | 0.4 | 1.10 | 1.01, 1.20 | 0.029 |
| BMI group |  |  |  |  |  |  |
| Healthy weight | — | — |  | — | — |  |
| Obese | 0.90 | 0.81, 0.99 | 0.032 | 0.89 | 0.83, 0.96 | 0.002 |
| Overweight | 0.97 | 0.89, 1.05 | 0.4 | 0.91 | 0.86, 0.96 | 0.001 |
| Underweight | 1.15 | 1.00, 1.32 | 0.046 | 1.40 | 1.28, 1.54 | <0.001 |
| Histology |  |  |  |  |  |  |
| Adenocarcinoma | — | — |  | — | — |  |
| Squamous cell | 1.22 | 1.12, 1.32 | <0.001 | 1.00 | 0.94, 1.06 | >0.9 |
| NSCLC, NOS | 1.04 | 0.90, 1.21 | 0.6 | 1.05 | 0.98, 1.12 | 0.2 |
| Other | 0.95 | 0.80, 1.11 | 0.5 | 1.06 | 0.96, 1.17 | 0.3 |
| Prior radiotherapy | 1.07 | 0.99, 1.15 | 0.084 | 1.00 | 0.95, 1.05 | >0.9 |
| Prior lines of systemic therapy | 1.09 | 0.99, 1.19 | 0.084 | 1.20 | 1.13, 1.29 | <0.001 |
| Charlson Comorbidity Index (excluding malignancies) |  |  |  |  |  |  |
| 0 | — | — |  | — | — |  |
| 1 | 1.13 | 1.02, 1.26 | 0.025 | 1.00 | 0.93, 1.07 | 0.9 |
| 2 to 3 | 1.08 | 0.97, 1.21 | 0.2 | 1.04 | 0.96, 1.12 | 0.4 |
| 4 to 6 | 1.12 | 0.98, 1.29 | 0.093 | 1.12 | 1.01, 1.24 | 0.026 |
| 7 or higher | 1.15 | 0.94, 1.40 | 0.2 | 1.10 | 0.90, 1.35 | 0.4 |
| Tumor PD-L1 expression |  |  |  |  |  |  |
| <1% | — | — |  | — | — |  |
| 1-49% | 0.89 | 0.80, 0.99 | 0.037 |  |  |  |
| 50-100% | 0.74 | 0.67, 0.82 | <0.001 |  |  |  |
| Unknown | 0.88 | 0.79, 0.98 | 0.020 |  |  |  |
| Smoking status |  |  |  |  |  |  |
| Never | — | — |  | — | — |  |
| Prior | 0.93 | 0.83, 1.03 | 0.2 | 0.96 | 0.88, 1.04 | 0.3 |
| Current | 0.87 | 0.77, 0.97 | 0.013 | 0.94 | 0.86, 1.02 | 0.13 |
| Unknown | 1.01 | 0.84, 1.21 | >0.9 | 0.95 | 0.86, 1.05 | 0.3 |
| COPD severity |  |  |  |  |  |  |
| No COPD | — | — |  | — | — |  |
| COPD, no exacerbations | 0.91 | 0.83, 0.99 | 0.030 | 0.93 | 0.87, 0.99 | 0.027 |
| COPD, 1-2 exacerbations | 1.00 | 0.89, 1.12 | >0.9 | 1.06 | 0.97, 1.15 | 0.2 |
| COPD, 3 or more exacerbations | 1.03 | 0.83, 1.29 | 0.8 | 0.94 | 0.77, 1.14 | 0.5 |
| ED visit in prior year | 1.17 | 1.08, 1.27 | <0.001 | 1.17 | 1.11, 1.23 | <0.001 |
| Social work visit in prior year | 1.15 | 1.07, 1.24 | <0.001 | 1.03 | 0.97, 1.08 | 0.3 |
| Mental health visit in prior year | 0.96 | 0.89, 1.04 | 0.3 | 0.93 | 0.88, 0.98 | 0.012 |
| Chronic kidney disease stage |  |  |  |  |  |  |
| 1 | — | — |  | — | — |  |
| 2 | 0.87 | 0.80, 0.94 | <0.001 | 0.92 | 0.87, 0.97 | 0.003 |
| 3a | 0.89 | 0.79, 1.01 | 0.082 | 0.98 | 0.89, 1.07 | 0.6 |
| 3b | 0.84 | 0.69, 1.02 | 0.079 | 0.95 | 0.82, 1.11 | 0.5 |
| 4 | 0.83 | 0.59, 1.17 | 0.3 | 1.14 | 0.84, 1.55 | 0.4 |
| 5 | 1.52 | 0.85, 2.73 | 0.2 | 0.76 | 0.44, 1.32 | 0.3 |
| Number of oncology visits in prior year | 1.00 | 0.99, 1.01 | 0.5 | 1.00 | 0.99, 1.01 | >0.9 |
| Baseline autoimmune disease (any) | 1.01 | 0.87, 1.18 | 0.9 | 1.05 | 0.95, 1.16 | 0.4 |
| Baseline hypothyroidism | 0.99 | 0.87, 1.11 | 0.8 | 0.96 | 0.87, 1.07 | 0.5 |
| ^1^HR = Hazard Ratio, CI = Confidence Interval | | | | | | |

Abbreviations: COPD: chronic obstructive pulmonary disease; ED: emergency department; NSCLC, NOS: non-small-cell lung cancer, not otherwise specified; PD-L1: programmed death ligand-1.
